# Impacts of Nucleosome Positioning Elements and Pre-Assembled Chromatin States on Expression and Retention of Transgenes

**DOI:** 10.3390/genes15091232

**Published:** 2024-09-21

**Authors:** Ronard Kwizera, Junkai Xie, Nathan Nurse, Chongli Yuan, Ann L. Kirchmaier

**Affiliations:** 1 Department of Biochemistry, Purdue University, West Lafayette, IN 47907, USA; rkwizera@purdue.edu; 2 Davidson School of Chemical Engineering, Purdue University, West Lafayette, IN 47907, USA; xie161@purdue.edu (J.X.); npnurse@gmail.com (N.N.)

**Keywords:** expression of transgenes, chromatin, histone acetylation, nucleosome positioning

## Abstract

Background/Objectives: Transgene applications, ranging from gene therapy to the development of stable cell lines and organisms, rely on maintaining the expression of transgenes. To date, the use of plasmid-based transgenes has been limited by the loss of their expression shortly after their delivery into the target cells. The short-lived expression of plasmid-based transgenes has been largely attributed to host-cell-mediated degradation and/or silencing of transgenes. The development of chromatin-based strategies for gene delivery has the potential to facilitate defining the requirements for establishing epigenetic states and to enhance transgene expression for numerous applications. Methods: To assess the impact of “priming” plasmid-based transgenes to adopt accessible chromatin states to promote gene expression, nucleosome positioning elements were introduced at promoters of transgenes, and vectors were pre-assembled into nucleosomes containing unmodified histones or mutants mimicking constitutively acetylated states at residues 9 and 14 of histone H3 or residue 16 of histone H4 prior to their introduction into cells, then the transgene expression was monitored over time. Results: DNA sequences capable of positioning nucleosomes could positively impact the expression of adjacent transgenes in a distance-dependent manner in the absence of their pre-assembly into chromatin. Intriguingly, the pre-assembly of plasmids into chromatin facilitated the prolonged expression of transgenes relative to plasmids that were not pre-packaged into chromatin. Interactions between pre-assembled chromatin states and nucleosome positioning-derived effects on expression were also assessed and, generally, nucleosome positioning played the predominant role in influencing gene expression relative to priming with hyperacetylated chromatin states. Conclusions: Strategies incorporating nucleosome positioning elements and the pre-assembly of plasmids into chromatin prior to nuclear delivery can modulate the expression of plasmid-based transgenes.

## 1. Introduction

The effective expression of transgenes for a variety of applications, including the generation of cell lines that stably express a protein of interest for basic research or industrial purposes related to gene therapy, relies on the long-term expression of transgenes at appropriate levels. Currently, gene therapy strategies are predominantly applied through engineered integrating or non-integrating viral vehicles that are based on adenovirus, adeno-associated virus, or retroviruses [1,2,3,4,5]. However, several challenges accompany viral-based gene therapy, including immunogenicity and the cost [4,6,7]. Moreover, when transgene vectors become integrated into the host-cell genome, their transgenes may be subject to position-effect variegation where chromatin states adjacent to sites of integration are adopted, often resulting in the transgene becoming silenced [8,9,10]. Such issues motivate the development of alternate approaches, including the application of nonviral plasmid-based vectors. However, one major challenge limiting the effective use of nonviral plasmids has been the rapid loss of expression of the encoded transgene following delivery into cells and organisms [11,12,13]. This can occur through rapid loss of the plasmid DNA itself from proliferating cell populations in the absence of selection [14,15,16,17], or through the adoption of chromatin states that are incompatible with efficient gene expression [18,19,20]. Like the host-cell genome, exogenous episomal DNAs delivered into cells undergo host-cell-mediated packaging into chromatin and acquire post-translational modifications in that chromatin that can either promote or silence the expression of transgenes over time [21,22].

The transcription of DNA within chromatin is influenced by post-translational modifications made to histones, carried out by controlling the accessibility to DNA promoter regions via the relaxation or compaction of chromatin or the via recruitment of transcriptional machinery [23,24,25,26,27]. For example, the acetylation of lysine residues on H3 and H4 neutralizes their positive charges, thus reducing their affinity for the negatively charged DNA, and thereby relaxing nucleosomes in acetylated chromatin [28,29]. Histone acetylation correlates with active gene expression, whereas deacetylation, which restores positive charges to lysine residues, correlates with low levels of gene expression or gene silencing [30,31]. Consistent with this trend, acetylated H3K9 and H3K14 are enriched at promoters of actively transcribed genes [32,33]. Also, acetylated H4K16 promotes the formation of relaxed chromatin via disrupting inter-nucleosome interactions and, thus, can enhance accessibility of DNA within chromatin [29]. In contrast to acetylation, other modifications promote the silencing of gene expression. For example, the methylation of H3K9 by SUV39H1 both prevents H3K9 acetylation and promotes gene silencing via serving as a binding site for HP1, a structural component of heterochromatin [24,34,35,36].

The successful long-term application of nonviral, plasmid-based transgene vectors will require the establishment and maintenance of chromatin states that promote access of the transcriptional machinery to regulatory elements at genes and the prolonged expression of those transgenes. Several strategies aimed at overcoming challenges associated with the delivery and expression of transgenes have been explored previously, including the application of enhancer elements to facilitate expression [37,38,39], or of insulator elements (e.g., ubiquitous chromatin opening elements (UCOEs) and stabilizing anti-repressor elements (STARs)) to protect transgenes from host-cell-mediated silencing [40,41,42]. While insulator-based strategies can improve transgene expression, they have also been associated with a decline in expression following prolonged culturing and dramatic decreases in vector copy numbers shortly after delivery [43,44], possibly due to increased transgene expression causing toxicity [45,46,47]. The nuclear delivery of expression vectors can be enhanced via polyplexes with peptides based on the N-terminal tail of histone H3, which interacts with the nuclear import receptor Importin 4 [48,49,50]. The use of exogenous individual histone–plasmid DNA complexes has also been explored as a method of gene transfer, but has been limited in its success (for H3, see [51], see also [52]). However, in these systems, the charge–charge-based complexes formed between individual histones or histone fragments and DNA do not reflect the natural packaging of DNA into nucleosomes.

Here, we assessed how transgene expression from the human elongation factor 1a (EF1α) promoter was impacted by the introduction of nucleosome positioning elements as well as the pre-assembly of the nucleosomes using recombinant hypoacetylated wild-type histones or, to promote accessibility, histones containing lysine (K) to glutamine (Q) mutations to mimic acetylated states at histone H3 K9,14 or histone H4 K16 prior to their delivery into cells. By monitoring the transient and prolonged expression of the reporter-enhanced green fluorescent protein, eGFP, we observed that the inclusion of nucleosome positioning sequences as well as the pre-assembly of the reporter plasmid into chromatin prior to delivery into cells impacted the efficiency of the transient or prolonged expression of eGFP. Together, our observations indicated that strategies to promote chromatin accessibility through the introduction of nucleosome positioning elements adjacent to transgenes in plasmid-based vectors, or through the pre-assembly of vectors into chromatin containing attributes of active chromatin states, have the potential to enhance transgene expression in human cells.

## 2. Materials and Methods

### 2.1. Cloning of Reporter Plasmids

**Figure 1:**
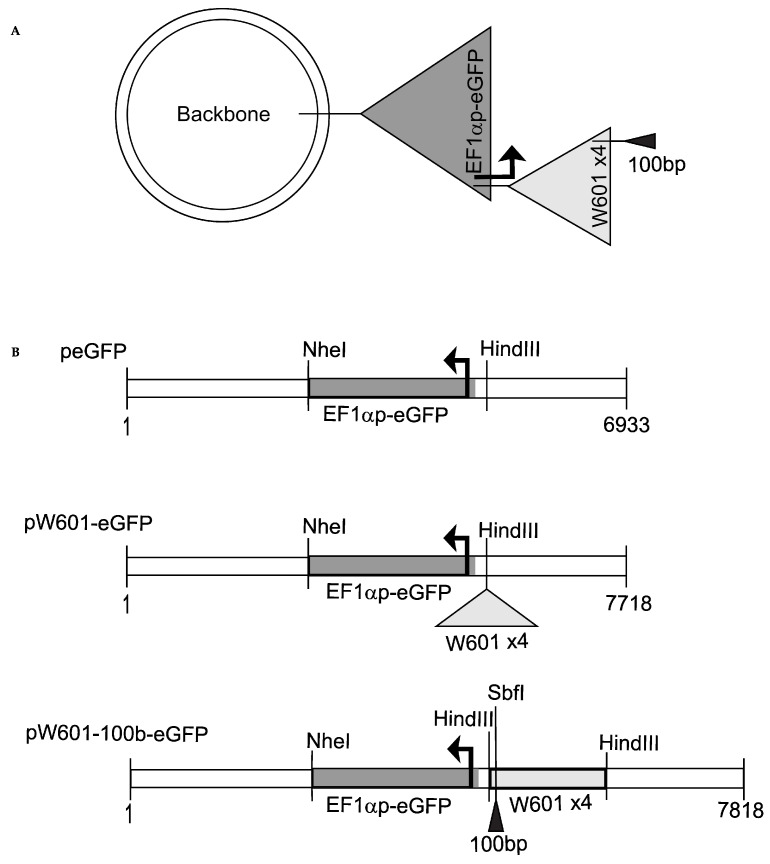
Reporter plasmids used in this study. (**A**) Cloning strategy. A plasmid backbone containing beta-lactamase and aminoglycoside 3′-phosphotransferase (APH (3′)) genes, and bacteria origin of replication (ori) was used as the parent plasmid for cloning eGFP reporter plasmids (see Materials and Methods); (**B**) organization of plasmids peGFP, pW601-eGFP, and pW601-100b-eGFP. peGFP contains the eGFP gene expressed from the human EF1α promoter (sequence in Figure S1A). pW601-eGFP contains an array of four 147 bp direct repeats of the Widom601 nucleosome positioning sequence with a 30 bp linker sequence between each repeat, cloned into a HindIII site of peGFP. pW601-100b-eGFP contains a 100 bp fragment cloned into the SbfI site of pW601-eGFP. Genes and other sequences are not drawn to scale.

The plasmid peGFP, expressing the enhanced green fluorescence protein (eGFP) under the control of the human Elongation Factor 1 Alpha (EF1α) promoter [53,54] (Figure S1A), was used to generate plasmids pW601-eGFP and pW601-100b-eGFP. The E1Fα promoter was chosen, as this promoter prolongs transgene expression without affecting the vector copy number [55], whereas the commonly used cytomegalovirus promoter (CMVp) is prone to epigenetic silencing [56,57]. To generate pW601-eGFP, an array of four direct repeats of a 177 bp sequence that contained the 147 bp Widom601 sequence, which is a SELEX-selected DNA sequence that has high affinity for histone octamers and can efficiently position nucleosomes [58], was amplified by PCR from plasmid pUC19-Widom601 [59] using primers oALK1723/1724 (5′ GCGAAGCTTGAATTCGAGCTCGGTACCCG 3′/5′ GCCAAGCTTGCATGCCTG 3′, Integrated DNA Technologies (IDT)). The amplified Widom601 fragment was then digested with HindIII (New England Biolabs (NEB), Ipswich, MA, USA) and gel-isolated using the QIAquick Gel Extraction Kit (50) (Qiagen, Hilden, Germany). Purified products were cloned into the unique HindIII site of peGFP (Figure 1). To generate pW601-100b-eGFP, a 100-base pair fragment was amplified from pUC19 (#50005, Addgene) (Figure S1B) using primers oALK1765/1766 (5′ GCTTGCATGCCTGCAGGCCTGGGGTGCCTAATGAG 3′/5′ CGTCTTCGATATCCTGCATAATGCAGCTGGCACGAC 3′, IDT), then digested with SbfI (NEB) and gel-isolated. Isolated products were cloned into the unique SbfI site of pW601-eGFP using NEBuilder^®^ HiFi DNA Assembly Master Mix (#E2621S, NEB). Plasmids were validated by restriction enzyme digestion using HindIII or SbfI-HF and sequenced using primers oALK1727/oALK1728 (5′ ACCGGTTCAATTGCCGAC 3′/5′ CCACAACTAGAATGCAGTG 3′, IDT). p100-eGFP was generated from pW601-100b-eGFP by deleting Widom601 array using EcoRV (NEB) (Figure S1A). Expression of eGFP was tested by epifluorescence microscopy analyses in transient expression assays. Plasmids were isolated from DH5α *E. coli* under endotoxin-free conditions using the PureLink HiPure Plasmid DNA Purification Kits (K2100-02, Invitrogen, Waltham, MA, USA) prior to chromatin assembly.

### 2.2. Site-Directed Mutagenesis of H3K9,14 and H4K16 to Mimic Acetylated States

Plasmid pET21b, encoding histone H3 and H4 genes [60] from *Xenopus laevis* (*X. laevis*) was used to generate H3K9,14Q or H4K16Q mutants. The H3K9Q mutant was generated by site-directed mutagenesis using primers 5′ CGCCCGTCAGTCCACCGGAG 3′/5′ CCGGTGGACTGACGGGCGG 3′. Then, the H3 K9Q mutant was used to generate the H3K9,14Q mutant similarly using primers 5′ CGTAAATCCACCGGAGGGCAGGCTCCCCGCAAGCAGC 3′/5′ GCTGCTTGCGGGGAGCCTGCCCTCCGGTGGATTTACG 3′. The H4K16Q mutant was generated similarly using primers 5′ GGGTAAAGGTGGTGCTCAGCGTCACCGTAAAGTTC 3′/5′ GAACTTTACGGTGACGCTGAGCACCACCTTTACCC 3′. Mutated sequences were verified via Sanger sequencing.

### 2.3. Refolding of Histone Octamers and Assembly of Chromatin

Recombinant core histones (H2A, H2B, H3, H4) or mutants (H3K9,14Q and H4K16Q) from *X. laevis* were expressed and purified from *E. coli* (BL21 (DE3)) as described in our previous studies [59,61]. The homology between human and *X. laevis* H4 is 100%, that of H3 is 97%, and the H3–H4 N terminal tail regions as well as H4 K16 and H3 K9,14 are conserved. To assemble plasmids into chromatin while mimicking the length of DNA in a nucleosome [58,62,63], recombinant unmodified histone octamers were mixed with plasmids at a molar ratio of 0.3 moles of histone octamers per every 177 bp DNA length, corresponding to 147 bp nucleosomal DNA plus a 30 bp linker DNA sequence, as cloned in the Widom601 array-containing plasmids. Mixed samples were reconstituted into chromatin under salt gradient dialysis as described in other studies [60,64,65]. Successful and efficient assembly was validated by DNA gel electrophoresis, see Figure S2 for representative validation analysis that confirmed all detectable plasmid had been assembled into chromatin.

### 2.4. Transfection by Calcium Phosphate

A total of 5 × 10^4^ 143B (CRL-8303, ATCC) cells were seeded per well on a 6-well plate for 24 h prior to transfection. A total of 0.6 picomole of plasmids were pre-assembled into chromatin or unassembled plasmid DNA, and a final culture concentration of 11.5 mM CaCl_2_ was used per transfection. Briefly, a 230 μL mixture was generated for each transfection by adding 2X HEPES-Buffered Saline (HBS) with a pH of 7.0 containing 50 mM HEPES, 280 mM NaCl, and 1.5 mM Na_2_HPO_4_ to a final concentration of 1X HBS, followed by DNA and, lastly, 100 mM CaCl_2_. The mixture was allowed to incubate at room temperature for 10 min to allow formation of calcium-phosphate precipitates prior to adding to cell cultures [66]. Twenty-four hours post-transfection, cell supernatant was removed, and cells were supplemented with fresh growth media containing DMEM, 10% fetal bovine serum, and 100 units of penicillin-streptomycin. Cultures were maintained in 37 °C incubator containing 5% CO_2_. Lipofectamine-based transfection was not used in these analyses as cationic lipids and positively charged histones will compete for negatively charged DNA.

### 2.5. Flow Cytometry Analyses of Expression of Reporter eGFP

Untransfected 143B cells or 143B cells transfected with plasmids that encoded the reporter eGFP were trypsinized with 1X Trypsin-EDTA (0.5%), aliquots of cells were stained with 0.4% trypan blue and counted using a hemocytometer, and 1 × 10^5^ viable cells were isolated by centrifugation at 300× *g* for 3 min at room temperature. Cells were resuspended in 1X phosphate-buffered saline (1X PBS) containing 137 mM NaCl, 2.7 mM KCl, 10 mM Na_2_HPO4, and 1.8 mM KH2PO4, pH 7.4. Cell pellets were resuspended in 200 μL 1X PBS and kept on ice until flow cytometry. Samples were filtered through cell strainer FACS tubes (Stellar Scientific, Baltimore, MD, USA, FSC-9005) to remove clumped cells just prior to flow cytometry analysis. Samples were analyzed using a BD Accuri C6 Plus flow cytometer and BD Accuri C6 Plus software, version 1.0.27.1. The expression of reporter eGFP was analyzed using the FL-1 488 nm channel. Mean fluorescence intensity in the FL-1 488 nm channel below 1 × 10^4^ was set to background in this study. Untransfected cells served as the negative control. See Supplemental Figure S3 for representative epifluorescence microscopy images and example of flow cytometry analysis strategy used herein.

### 2.6. Statistical Analysis of Expression of Reporter eGFP

One-way ANOVA [67] was used to compare the individual means of the percent of eGFP+ cells from a given plasmid construct to the overall mean of percent of eGFP+ cells from all plasmids within an experiment. One-way ANOVA was also used to compare mean fluorescence intensity of those cells expressing eGFP from a given plasmid construct to the overall mean of all plasmids tested. To obtain *p* values, Tukey’s honestly significant difference (TukeyHSD) [68] post-hoc test was used to perform pairwise comparisons of eGFP expression between plasmid constructs. *p* values < 0.05 were considered significant.

### 2.7. Rate of Loss of Expression of Reporter eGFP

To determine the rate of loss of cells expressing eGFP from the total cell population, or the rate of loss of fluorescence intensity from the eGFP+ subpopulation, the number of cell generations at every time point from Day 3 (D3) to Day 9 (D9) was calculated as the number of hours after transfection divided by the cell doubling time (Equation (1)). The doubling time was computed as the number of hours after transfection times log (2) divided by the difference between log of final cell count, at the time of analysis, and log of initial cell count, at the time of seeding (Equation (2)). For the rate of loss of percent of eGFP+ cells or the fluorescence intensity, the slope (m) of the linear regression line, Y = mX + b, where Y was percent eGFP+ cells or fluorescence intensity, X was the number of cell generations, and b was the Y-intercept (Equation (3)), was computed.

Number of cell generations = hours after transfection/cells doubling time(1)

Doubling time = [(hours after transfection) × log (2)]/[log (final cell count) − log (initial cell count)](2)

Rate of loss (m) = (Y − b)/X(3)

## 3. Results

### 3.1. Cis-Sequences Impact the Efficiency of Expression of eGFP from Plasmids Transfected as Naked DNA

In mammals, ~147 bp of negatively charged DNA is wrapped around a positively charged histone octamer to form a nucleosome through electrostatic interactions, and adjacent nucleosomes are separated by inter-nucleosomal “linker DNA” within chromatin [69]. Certain DNA sequence motifs such as the artificially derived Widom601 sequence have high affinity for histone octamers and can ‘position’ the octamer at these motifs as well as promote the positioning of neighboring nucleosomes into ordered, or phased, arrays upon nucleosome assembly both in vitro and in vivo [58,70,71,72]. To establish the impact of *cis*-sequences capable of promoting nucleosome positioning on the efficiency of the expression and retention of reporter eGFP, we generated a series of reporter plasmids encoding the eGFP expressed from the human EF1α promoter [53,54,73] (Figure 1 and Figure S1). Four 177 bp direct repeats containing the Widom601 sequence [58,70] were cloned upstream of the EF1α promoter (Figure 1) to drive the assembly of positioned nucleosomes and to promote the phasing of nucleosomes across adjacent DNA sequences [74]. The TATA box-containing EF1α promoter has several regulatory regions that facilitate efficient promoter activity [53,54] (Figure S1A). As nucleosome phasing adjacent to nucleosomes positioned by the Widom601 array had the potential to affect the accessibility of *cis*-acting regulatory elements within the EF1α promoter, a third construct containing 100 bp of fragment cloned between the Widom601 array and the EF1α promoter was also generated to maximize the shift in nucleosome phasing across the promoter region, as 100 bp is approximately one half of the unit length of one nucleosomal DNA plus one linker DNA. This introduced fragment lacks eukaryotic DNA and did not enhance the transcription from the EF1α promoter (Figure S4). Of note, we and others have previously established that plasmids that are related to those described here and that lack the EBV latent origin of DNA replication do not replicate in the human osteosarcoma cell line 143B (e.g., [75,76]). Thus, the effects on transgene expression reported in this study are plasmid replication-independent.

**Figure 2:**
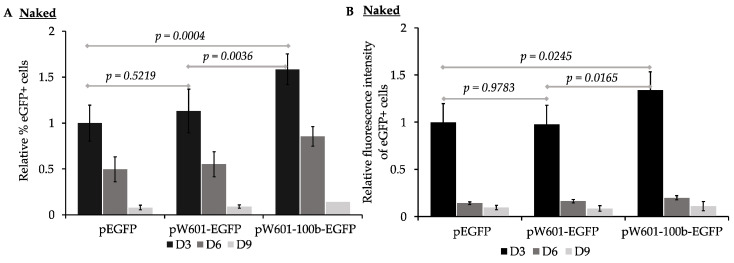
Nucleosome positioning sequences impact the efficiency of expression of eGFP from plasmids lacking pre-assembled nucleosomes. 143B cells transfected with the indicated plasmids (lacking pre-assembled nucleosomes) were analyzed for expression of eGFP by flow cytometry at ~72 h (D3), ~144 h (D6), and ~216 h (D9) post-transfection. (**A**) Percent of eGFP+ cells at D3, D6, and D9. Percentages of eGFP+ cells were normalized relative to percentages of eGFP+ cells transfected with peGFP at D3, which was set to 1 (% eGFP+ cells of indicated sample at D3, D6, or D9/% eGFP+ cells transfected with peGFP at D3; mean ± STD, *n* = 6); (**B**) mean fluorescence intensity of the eGFP+ subpopulations in (**A**) were determined for each sample and timepoint, and then normalized relative to the mean fluorescence intensity of cells transfected with peGFP at D3, which was set to 1 (fluorescence intensity of indicated sample at D3, D6, or D9/fluorescence intensity of cells transfected with peGFP at D3; mean ± STD, *n* = 6). Statistical analyses were conducted using one-way ANOVA, and indicated *p* values were calculated using the TukeyHSD post-hoc test.

To assess the impact of nucleosome positioning sequences on the efficiency of the expression and retention of reporter eGFP from plasmids transfected as “naked DNA”, the parent plasmid (peGFP), the plasmid containing Widom601 array (pW601-eGFP), or the plasmid containing the Widom601 array plus a 100 bp insert (pW601-100b-eGFP) (Figure 1B) were transfected into human 143B cells and assessed for the efficiency of expression of eGFP at ~72 h (D3), 144 h (D6), and 216 h (D9) post-transfection by flow cytometry. The expression of eGFP was assessed by two parameters: the percentage of cells in the population that expressed eGFP, as well as the intensity of the fluorescence of the eGFP+ subpopulation (Figure 2). eGFP was expressed in 15.8 ± 4.6%, *n* = 6, of the cells transfected with peGFP at three days post-transfection. This eGFP+ subpopulation had a mean fluorescence intensity of 890,541 ± 68,348, *n* = 6. Introduction of the Widom601 array upstream of the EF1α promoter (pW601-eGFP) did not significantly impact the percentage of cells transiently expressing eGFP relative to those cells transfected with the parent plasmid peGFP (Figure 2A). In contrast, transfection with pW601-100b-eGFP resulted in a significantly greater percentage of eGFP+ cells relative to that observed for either peGFP or pW601-eGFP at three days post-transfection, *p =* 0.0004 and *p =* 0.0036, *n* = 6, respectively. Similarly, the eGFP+ subpopulation in cells transfected with pW601-100b-eGFP exhibited a higher mean fluorescence intensity relative to that observed in cells transfected with peGFP or pW601-eGFP at three days post-transfection, *p =* 0.0245 and *p =* 0.0165, *n* = 6, respectively (Figure 2B). Together, these observations indicated that sequences capable of influencing nucleosome positioning could alter the percentage of cells initially expressing eGFP as well as the level of eGFP expressed in those cells.

GFP has a half-life (~26 h [77]) that is similar to the doubling time of 143B cells (24 h [75,78]). For cells to express eGFP, at least one transcriptionally active copy of the transgene plasmid must be present, or recently present, in the cells. To assess whether *cis*-elements capable of promoting nucleosome positioning impacted the short-term retention of the expression of eGFP, the rate of loss of eGFP+ cells per generation from the transfected populations was determined for cells that had been transfected with plasmids that lacked (peGFP) or contained nucleosome positioning sequences (pW601-eGFP and pW601-100b-eGFP) (Table 1). Over the course of nine days, the eGFP+ cells were lost at similar rates from the cell populations transfected with either peGFP or pW601-eGFP, whereas the cells transfected with pW601-100b-eGFP lost their eGFP+ subpopulation more rapidly relative to those transfected with peGFP, *p =* 0.0066, *n* = 6 (Table 1). The intensity of the eGFP expression within eGFP+ cells can be influenced by factors such as the number of transcriptionally active transgene plasmids in a cell, as well as the levels of transcriptional activity and bursting [79]. In contrast to the rate of loss of eGFP+ cells, no significant difference was observed in the rate of loss of the intensity of fluorescence from the eGFP+ subpopulations over time, regardless of whether the cells had been transfected with plasmids containing or lacking nucleosome positioning sequences (Table 1). Together, these results are consistent with *cis*-sequences capable of altering nucleosome phasing influencing the retention of the expression of transgenes over time.

### 3.2. Pre-Assembled Chromatin Retain Expression of eGFP more Efficiently than Naked Plasmid DNA

**Figure 3:**
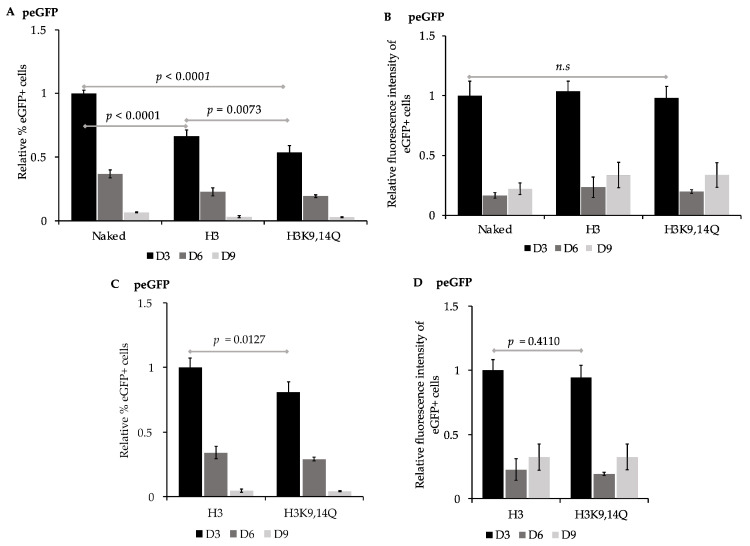
Impacts of pre-assembling peGFP into chromatin on expression of eGFP. 143B cells transfected with peGFP (unassembled), or peGFP pre-assembled with unmodified recombinant histone octamers (H3) or with histone octamers containing H3K9,14Q were analyzed for expression of eGFP by flow cytometry at ~72 h (D3), ~144 h (D6), and ~216 h (D9) post-transfection. (**A**) Percent of eGFP+ cells at D3, D6, and D9 were calculated for samples transfected with the indicated constructs, and then normalized relative to percent of eGFP+ cells at D3 from cells transfected with (naked) peGFP, which was set to 1 as in Figure 2 (mean ± STD, *n* = 4); (**B**) mean fluorescence intensities of eGFP+ subpopulations in (**A**) were calculated and then normalized relative to the mean fluorescence intensity at D3 of cells transfected with (naked) peGFP, which was set to 1 (see Figure 2, mean ± STD, *n* = 4); (**C**,**D**) impact of H3 vs. H3K9,14Q-containing chromatin on expression of eGFP. (**C**) Percentages of eGFP+ cells at D3, D6, and D9 from cells transfected with the peGFP pre-assembled with histone octamers containing H3 (H3) or H3K9,14Q (H3K9,14Q) were calculated and then normalized relative to percent of eGFP+ cells at D3 from cells transfected with peGFP pre-assembled with unmodified histones (H3), which was set to 1 (mean ± STD, *n* = 4). (**D**) Mean fluorescence intensity of eGFP+ subpopulation in (**C**) was normalized relative to mean fluorescence intensity of eGFP+ subpopulation at D3 from cells transfected with peGFP pre-assembled with unmodified histone octamers (H3), which was set to 1 (mean ± STD, *n* = 4). Statistical significance and *p* values were determined as in Figure 2.

The accessibility of regulatory sequences within nucleosomal DNA by transcription machinery can be facilitated via the post-translational modification of histones to disrupt charge–charge interactions between the histone tails and DNA. Transcriptionally repressed regions and condensed chromatin are enriched with deacetylated histones [30,31,80,81]. In contrast, the acetylation of lysine residues in histone tails, which neutralizes their positive charge, is associated with an enhanced accessibility of DNA and gene expression [28,29,82,83], and transcriptionally active regions are enriched with acetylated histones including acetylated histone H3 at K9 [33] and K14 [33,84], and histone H4 at K16 [29,85]. Consistent with histone acetylation states influencing the expression from the EF1α promoter, treatment with either Class I or Class II histone deacetylase inhibitors at the time of transgene delivery via plasmid vectors enhances the transgene expression from the EF1α promoter [86,87,88]. To assess the impact of different pre-assembled chromatin states on the efficiency of expression of eGFP, peGFP was first pre-assembled into nucleosomal DNA in vitro using histone octamers that contained either the recombinant unmodified histones H2A, H2B, H3, and H4 or histone octamers containing H2A, H2B, H3K9,14Q, and H4 (Figure 3), as the neutral charge of acetylated lysine residues can be mimicked by mutating lysine (K) to glutamine (Q) [82,83]. “Naked” peGFP or pre-assembled constructs were then transfected into 143B cells, and the percent of eGFP+ cells and the intensity of fluorescence of those eGFP+ cells were evaluated as in Figure 2. eGFP was expressed in 15.7 ± 0.4%, *n* = 4, of the cells transfected with peGFP lacking pre-assembled nucleosomes at three days post-transfection. The mean fluorescence intensity of this eGFP+ subpopulation was 430,944 ± 52,314, *n* = 4. In contrast, cells transfected with peGFP that had been pre-assembled with either H3- or H3K9,14Q -containing histone octamers had fewer eGFP+ cells in their population at three days post-transfection relative to the cells transfected with naked peGFP, *p* < 0.0001 and *p* < 0.0001, *n* = 4, respectively (Figure 3A). The percentage of eGFP+ cells in the cells transfected with peGFP that had been pre-assembled into chromatin with unmodified H3-containing octamers prior to transfection was greater than that of those assembled into chromatin with octamers containing H3K9,14Q at three days post-transfection, *p* = 0.0127, *n* = 4 (Figure 3C). However, the mean fluorescence intensity of the eGFP+ subpopulations from all conditions tested were similar at three days post-transfection (Figure 3D). Together, these observations indicate that pre-assembled chromatin states could impact the percentage of cells that initially expresses transgenes; the pre-assembly of unmodified, “deacetylated” histones reduced the initial levels of expression of eGFP, but mimicking the acetylated state on residues 9 and 14 of H3 in this context was insufficient to promote gene expression. However, the assembled chromatin states did not alter the initial level of expression of eGFP within the eGFP+ subpopulations relative to that of the naked peGFP.

Next, we determined the rate of loss of the expression of eGFP in cells transfected with naked peGFP or with peGFP pre-assembled into chromatin containing either unmodified H3 or H3K9,14Q (Figure 3 and Table 2). Cells transfected with peGFP pre-assembled into unmodified nucleosomes retained their expression of eGFP more efficiently than those transfected with naked peGFP, *p* = 0.0012, *n* = 4 (Table 2). Similarly, cells transfected with peGFP pre-assembled into nucleosomes containing H3K9,14Q retained their expression of eGFP more efficiently than naked peGFP, *p* = 0.0008, *n* = 4, or peGFP assembled into unmodified nucleosomes, *p* = 0.0141, *n* = 4. In contrast, no differences in the retention of the intensity of the eGFP fluorescence signal over time were observed in those eGFP+ subpopulations across all constructs (Table 2). Together, these observations imply that the delivery of reporter plasmids pre-assembled into different forms of chromatin can influence both the initial and continued expression of transgenes relative to naked gene delivery.

### 3.3. Nucleosome Positioning Elements Affect the Efficiency of Expression of Reporter eGFP in Pre-Assembled Chromatin States

**Figure 4:**
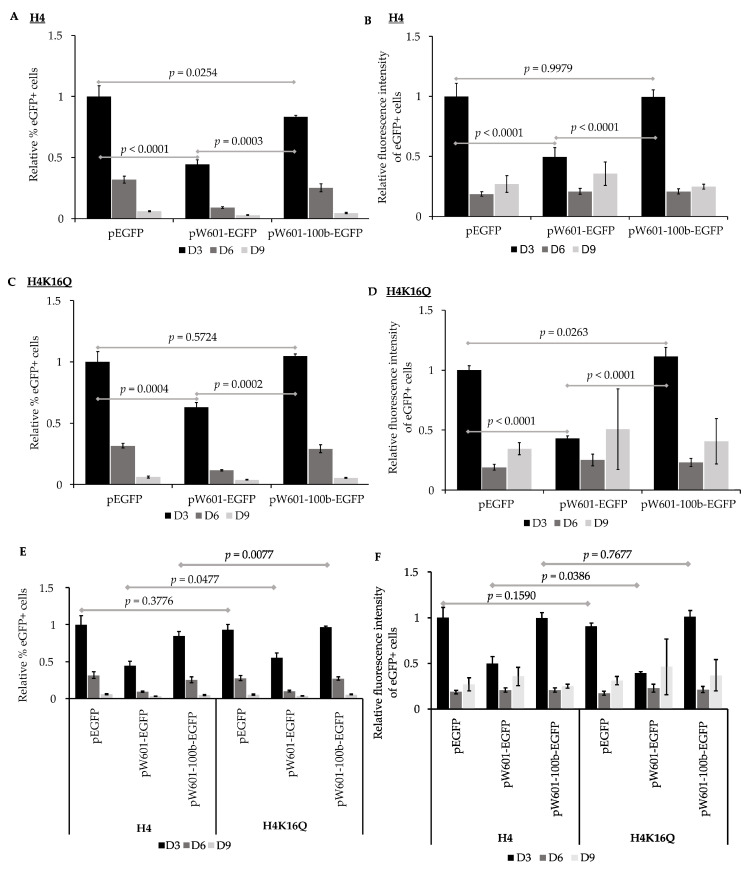
The impact of nucleosome positioning and pre-assembled chromatin states on expression of eGFP. 143B cells transfected with indicated plasmids pre-assembled with unmodified recombinant histone octamers (H4) or pre-assembled with histone octamers containing H4K16Q were analyzed for expression of eGFP by flow cytometry at ~72 h (D3), ~144 h (D6), and ~216 h (D9) post-transfection. (**A**) Percentages of eGFP+ cells at D3, D6, and D9 were calculated for samples transfected with the indicated plasmids pre-assembled with unmodified histone octamers (H4). Percent of eGFP+ cells was then normalized relative to percent of eGFP+ cells at D3 from cells transfected with peGFP pre-assembled with unmodified histone octamers, which was set to 1 (mean ± STD, *n* = 4), as in Figure 2; (**B**) mean fluorescence intensity of eGFP+ subpopulation in (**A**) was calculated and then normalized relative to mean fluorescence intensity at D3 from cells transfected with peGFP pre-assembled with unmodified histone octamers, which was set to 1 (mean ± STD, *n* = 4), as in Figure 2; (**C**) percent of eGFP+ cells at D3, D6, and D9 was calculated for samples transfected with the indicated plasmids pre-assembled with histone octamers containing H4K16Q. Percent of eGFP+ cells was then normalized relative to percent of eGFP+ cells at D3 from cells transfected with peGFP pre-assembled with H4K16Q histone octamers, which was set to 1 (mean ± STD, *n* = 4); (**D**) mean fluorescence intensity of eGFP+ subpopulation in (**C**) was calculated and then normalized relative to mean fluorescence intensity at D3 from cells transfected with peGFP pre-assembled with H4K16Q, which was set to 1 (mean ± STD, *n* = 4); (**E**,**F**) impacts of *cis*-elements and pre-assembled chromatin states on expression of eGFP. (**E**) Percent of eGFP+ cells at D3, D6, and D9 from cells transfected with the indicated plasmids pre-assembled with unmodified histone octamers (H4) or those containing H4K16Q (H4K16Q) (**A**,**C**) were re-analyzed by normalizing relative to percent of eGFP+ cells at D3 from cells transfected with peGFP pre-assembled with unmodified histone octamers (H4), which was set to 1 (mean ± STD, *n* = 4). (**F**) Mean fluorescence intensities of each eGFP+ subpopulation (**B**,**D**) were re-analyzed by normalizing relative to mean fluorescence intensity of the eGFP+ subpopulation at D3 from cells transfected with peGFP pre-assembled with unmodified histone octamers (H4), which was set to 1 (mean ± STD, *n* = 4). Statistical significance and *p* values were determined as described in Figure 2.

Nucleosome positioning can influence the expression state of endogenous genes [69,89,90,91]. To assess the impact of nucleosome positioning on the expression of transgenes from plasmids that had been pre-assembled into chromatin prior to delivery into cells, peGFP, pW601-eGFP, or pW601-100b-eGFP were initially pre-assembled into nucleosomes containing recombinant unmodified histones, then transiently transfected into 143B cells. The efficiency of the expression of eGFP was evaluated by determining both the percent of eGFP+ cells as well as the intensity of eGFP fluorescence in the eGFP+ subpopulation (Figure 4A,B), as shown above. When cells were transfected with unmodified nucleosomal peGFP, eGFP was expressed in 23.8 ± 3% of the cells at three days post-transfection, and this eGFP+ subpopulation had a mean fluorescence intensity of 402,776 ± 44,433, *n* = 4. Introduction of the Widom601 array upstream of the transcriptional start site at the EF1α promoter (pW601-eGFP) reduced the percentage of eGFP+ cells at three days post-transfection relative to that for the cells transfected with unmodified nucleosomal peGFP, *p* < 0.0001, *n* = 4 (Figure 4A). In contrast, insertion of the 100 bp fragment between the Widom601 array and the EF1α promoter to shift nucleosome phasing (pW601-100b-eGFP) partially suppressed the Widom601-dependent defects (pW601-eGFP) observed in the percent of eGFP+ cells at three days post-transfection, *p* = 0.0003, *n* = 4 (Figure 4A). However, the percentage of eGFP+ cells was not fully restored to the levels observed for cells transfected with unmodified nucleosomal peGFP at three days post-transfection, *p* = 0.0254, *n* = 4 (Figure 4A). Inclusion of the Widom array (pW601-eGFP) similarly reduced the mean fluorescence intensity of the eGFP+ subpopulation at three days post-transfection relative to that of either the peGFP or pW601-100b-eGFP, *p* < 0.0001 and *p* < 0.0001, *n* = 4, respectively (Figure 4B). In contrast, the mean fluorescence intensity of the eGFP+ subpopulations from cells transfected with peGFP or pW601-100b-eGFP were similar (Figure 4B). When comparing the retention of eGFP expression as a function of time from plasmids that had been pre-assembled into nucleosomes with unmodified histones, cells that expressed eGFP from pW601-eGFP retained their expression more efficiently than did cells transfected with peGFP or pW601-100b-eGFP, *p* = 0.0004 and *p* = 0.0002, *n* = 4, respectively (Table 3). Similarly, the eGFP+ subpopulation from pW601-eGFP retained the intensity of eGFP fluorescence more efficiently over time compared to those eGFP+ subpopulations from peGFP- and pW601-100b-eGFP-transfected cells, *p* = 0.0015 and *p* = 0.0005, respectively (Table 3). These observations highlight impacts of nucleosome positioning which influence the expression of transgenes from the E1Fα promoter.

The acetylation state of histone H4 K16 influences the chromatin structure, and the acetylated form of this residue promotes the formation of ‘relaxed’ chromatin [29]. Consistent with facilitating access to the *cis*-sequence in DNA, H4 K16 acetylation is enriched at the enhancers and transcriptional start sites of active genes [92]. To assess the impact of H4 K16 acetylation, or H4 K16 acetylation plus nucleosome positioning, on the efficiency of expression of eGFP, histone octamers containing the acetyl mimic H4K16Q were also used to pre-assemble nucleosomes onto peGFP, pW601-eGFP, and pW601-100b-eGFP (Figure 4C,D) as part of the experiment shown in Figure 4A,B. For 143B cells transfected with peGFP pre-assembled into H4K16Q-containing chromatin, GFP was expressed in 22.2 ± 1.6% of the cells at three days post-transfection. This eGFP+ subpopulation had a mean fluorescence intensity of 365,296 ± 14,127, *n* = 4. For the plasmids pre-assembled into H4K16Q-containing chromatin, fewer eGFP+ cells were observed in the 143B cells transfected with pW601-eGFP relative to either the amount in peGFP or pW601-100b-eGFP at three days post-transfection, *p* = 0.0004 or *p* = 0.0002, *n* = 4, respectively, whereas the percentage of eGFP+ cells was similar between peGFP and pW601-100b-eGFP (Figure 4C). Similarly, the presence of the Widom601 array in pW601-eGFP reduced the MFI of the eGFP+ subpopulation relative to that observed in the cells transfected with peGFP or pW601-100b-eGFP at three days post-transfection, *p* < 0.0001 and *p* < 0.0001, *n* = 4, respectively (Figure 4D). Further, the mean fluorescence intensity of this eGFP+ subpopulation was higher in cells transfected with pW601-100b-eGFP compared to that of the peGFP at three days post-transfection, *p* = 0.0263 (Figure 4D). Together, these observations are consistent with nucleosome positioning also affecting the expression from the E1Fa promoter when H4 K16Q nucleosomes are pre-assembled onto the reporter plasmids.

The data from this experiment (Figure 4A–D) were then re-analyzed to assess whether pre-packaging into nucleosomes containing H4K16Q would facilitate the expression of transgenes more efficiently than unmodified histones (Figure 4E,F and Figure S5). In this case, the samples were normalized relative to the percent of eGFP+ cells from cells transfected with peGFP pre-assembled with H4 (Figure 4E). Similarly, the mean fluorescence intensities of the eGFP+ subpopulations were normalized relative to the mean fluorescence intensity of the eGFP+ subpopulation from cells transfected with peGFP pre-assembled with H4 (Figure 4F). No difference in the percent of eGFP+ cells was observed for peGFP pre-assembled with histone octamers containing H4 relative to that of H4K16Q at three days post-transfection (Figure 4E and Figure S5A,D). In contrast, cells transfected with pW601-eGFP or pW601-100b-eGFP pre-assembled into H4K16Q-containing nucleosomes had a higher percentage of eGFP+ cells relative to those assembled into H4-containing nucleosomes, *p* = 0.0477 and *p* = 0.0077, respectively, *n* = 4, at three days post-transfection (Figure 4E). Despite increasing the percentage of cells that initially expressed eGFP under certain conditions, pre-assembly into H4 K16Q relative to H4-containing nucleosomes did not enhance the intensity of eGFP fluorescence from any of the plasmids at three days post-transfection (Figure 4F and Figure S5B,D,F). Similar to pre-assembly with H4-containing nucleosomes, cells transfected with pW601-eGFP pre-assembled into H4K16Q-containing nucleosomes retained eGFP+ cells in the population over time more efficiently than did cells transfected with similarly pre-assembled peGFP or pW601-100b-eGFP plasmids, *p* = 0.0005 and *p* = 0.0001, respectively (Table 4). Likewise, the eGFP+ subpopulation from pW601-eGFP pre-assembled into H4K16Q-containing nucleosomes retained the intensity of eGFP fluorescence over time more efficiently than similarly pre-assembled peGFP or pW601-100b-eGFP plasmids, *p* = 0.0155 or *p* = 0.0082, *n* = 4, respectively (Table 4). However, for all conditions, the fluorescence intensity of the eGFP+ cells initially decreased rapidly between three and six days post-transfection, then stabilized in the eGFP+ cells during later timepoints (Table 1, Table 2, Table 3 and Table 4). Thus, these data are also consistent with the Widom601 array influencing nucleosome assembly or positioning during pre-assembly in a manner that limited the maximal fluorescence intensity achieved in the eGFP+ cells at three days post-transfection.

## 4. Discussion

When naked plasmid DNA is delivered into cells, that DNA becomes assembled into chromatin by the host cell, and this can exert repressive or active effects on gene expression [8,20,21,93]. This study evaluated chromatin accessibility-based strategies, including nucleosome positioning and histone acetylation, for their application in establishing epigenetically transcriptionally active states of expression via “priming” the initial characteristics of chromatin at a transgene upon or prior to introduction into cells. Our observations indicated that an array of four Widom 601 nucleosome positioning elements [58,71] could negatively or positively influence the short-term expression of transgenes from plasmids in a manner dependent on their distance from the EF1α promoter when those plasmids were introduced into cells as naked DNA (Figure 2) or as pre-assembled chromatin (Figure 4). These observations were consistent with the host-cell-mediated packaging and phasing of nucleosomes on the reporter plasmids upon nuclear entry, as well as the pre-assembly of chromatin states, having impacted the accessibility of transcription machinery to the EFFα promoter to promote gene expression.

The EF1α promoter contains several regulatory sequences, including binding sites for the transcription factors Sp1 [94], Ap1 [95], EFP1, and EFP2 [54] (Figure S1), whose loss can negatively affect gene expression [53,54]. As shifting the distance between the EF1α promoter and the Widom 601 elements by 100 bp promoted the initial expression of eGFP, the initial positioning of nucleosomes across the EF1α promoter by the host cell may have influenced the transcription (Figure 3 and Figure 4) in a manner affecting *cis*-element accessibility or akin to position-effect variegation [96]. The observation that pW601-100b-eGFP exhibited a more rapid loss of eGFP+ cells over time relative to peGFP (Table 3 and Table 4) implied that, while nucleosome positioning could enhance the initial transgene expression, nucleosome positioning could also impact the stability of expression over time. Consistent with this, an initial high expression of transgenes is often associated with subsequent silencing, perhaps as a result of elevated cellular responses against the expression of foreign DNA [12,15].

Efficient gene expression is also facilitated by chromatin post-translational modifications, such as histone acetylation, that influence access to *cis*-acting regulatory elements by the transcription machinery at promoters [24,25,31]. In this study, the pre-assembly of peGFP with histone octamers containing either unmodified recombinant histones or H3K9,14Q mutants reduced the percentage of cells expressing eGFP at three days post-transfection relative to that of cells transfected with naked peGFP plasmid (Figure 3A). The effects of the unmodified histones were consistent with “priming” of the reporter via pre-assembly with hypoacetylated histone octamers having promoted a chromatin state that is incompatible with efficient gene expression. However, as acetylated H3K9 and H3K14 are enriched at promoters, and correlate with the activation of gene expression by RNA Polymerase II [32,33,97], it was somewhat surprising that “priming” with a hyperacetylated state at these residues on histone H3 was insufficient to promote the efficient production of eGFP+ cells. These differences in percentages of eGFP+ cells were not a result of cytotoxic effects of pre-assembled chromatin, as we observed no significant differences in cell growth or in the number of trypan blue-positive apoptotic cells [98]. Whether these differences also reflect difficulty in the cellular uptake of reporter plasmids that are pre-assembled into chromatin versus that of naked DNA by calcium phosphate-based transfection [66] and/or defects in delivery of the transgene vectors into the nucleus is currently unclear. Future optimization of delivery strategies for chromatinized vectors is warranted to clarify this issue.

While pre-assembled chromatin containing unmodified H3 or H3K9,14ac reduced the percentage of cells initially expressing eGFP, this effect was independent of the mean fluorescence intensity of eGFP observed within those eGFP+ cells (Figure 3). These observations were consistent with, on a cell-to-cell basis, pre-assembled H3 or H3 K9,14ac chromatin having adversely affected an early event such as the nuclear entry or initial recruitment of transcriptional machinery to the promoter, precluding the expression level that could be achieved. As the nuclear delivery of exogenous macromolecules via the nuclear pore is size-selective [99], and large molecules and proteins can be restricted to the cytosol until the nuclear envelope disassembles during mitosis [100,101], such factors may have influenced the efficiency of the nuclear delivery of chromatinized plasmids in this study. Also consistent with a nuclear import defect, H3K9,14Q lies in a region used by the nuclear import receptor Importin 4 to recognize unmodified H3 [102]. In the absence of nucleosome positioning (Widom601) sequences, in vitro nucleosome assembly likely occurred in a random manner that may have hindered the accessibility of *cis*-elements by transcription factors at the EF1α promoter (Figure S1A), thereby reducing the percentage of cells expressing eGFP (Figure 3). However, cells transfected with plasmids pre-assembled into H3K9,14Q-containing chromatin retained their expression of eGFP over time more efficiently than those transfected with naked plasmid DNA or plasmids pre-assembled with hypoacetylated histones (Table 2). Thus, pre-assembled acetylated states on H3 K9 and 14 promoted the prolonged expression of eGFP. The application of cell-tracking dyes such as Carboxyfluorescein diacetate succinimidyl ester (CFSE) should facilitate future studies on the contributions of pre-assembled chromatin to the retention of transgene expression across cell generations.

When evaluating the combined impacts of Widom601 nucleosome positioning elements and the pre-assembly of chromatin on plasmids prior to their introduction into cells, distance-specific effects of the Widom601 sequences on the expression of eGFP were observed. The presence of the Widom601 repeats led to the reduced expression of eGFP as measured by either the percent of eGFP+ cells or the fluorescence intensity of those eGFP+ cells when the reporter plasmid was pre-assembled into nucleosomal DNA containing unmodified histone octamers, relative to the absence of pre-assembly (Figure 2 and Figure 4). This may have reflected the nucleosome being positioned over regulatory elements at the promoter during assembly, as these effects could be suppressed by altering the distance between the Widom601 sequences and the promoter. Like for unmodified H4, pre-assembly with H4K16Q led to a Widom601-dependent and distance-specific reduction in the percent of eGFP+ cells and the efficiency of expression of eGFP in the eGFP+ cells (Figure 4). Such Widom601-derived effects on the expression of eGFP persisted even when the reporter was pre-assembled into a relaxed chromatin state (H4K16Q), despite H4K16ac being known to promote the formation of relaxed open or accessible DNA [28,29]. However, compared to chromatin containing unmodified H4, “priming” with H4K16Q-containing chromatin could mildly enhance eGFP expression (Figure 4E,F). Together, nucleosome positioning played the predominant role in influencing the expression from the EF1α promoter under the tested conditions. In the future, adjusting nucleosome positioning by varying the spacer lengths between the Widom601 array and the promoter may facilitate optimizing transgene expression in a promoter-specific manner, as the promoter strength is influenced by varying types and locations of *cis*-regulatory elements present in individual promoters. Moreover, assessing the effects reported here across multiple cell types should reveal the extent to which the pre-assembly of chromatin modulates cell-type-specific versus universal effects on gene expression.

In summary, our findings demonstrated that *cis*-sequences and pre-assembled chromatin states influenced the initial expression and retention of transgenes, and provide a foundation for the further development of chromatin-based strategies to increase the probability of forming epigenetically active states of expression upon the delivery of transgenes into target cells. The ability to modulate nucleosome positioning and chromatin post-translational modifications has the potential to provide a powerful toolkit for multiple potential applications, including gene therapy, where precise control over gene expression is critical. Future studies assessing the impacts of additional modifications, either singly or in combination, together with *cis*-acting elements should further refine the control of the expression of transgenes. Circumventing potential difficulties in the nuclear delivery of pre-assembled chromatin relative to that of naked DNA may be possible via introducing nuclear targeting sequences to facilitate their nuclear entry [48,49,50]. Further, combining the novel chromatin-based strategy described herein with other approaches, including the addition of non-B DNA elements [103,104,105] or matrix attachment regions [106] may also facilitate optimizing the expression of nonviral plasmid-based transgenes in the future.

## Figures and Tables

**Table 1:** Loss of expression of eGFP from reporter plasmids lacking pre-assembled nucleosomes per cell generation.

Unassembled Plasmids	% Loss of eGFP+ Cells/Generation ^a^	% Loss of Fluorescence Intensity/ Generation ^a^
peGFP	18.4 ± 4 ^b^	17 ± 4 ^e^
pW601-eGFP	20.3 ± 5 ^c^	15.4 ± 4 ^f^
pW601-100b-eGFP	25.7 ± 3 ^d^	22.2 ± 7.6 ^g^

^a^ *n* = 6; ^b,c^ *p* = 0.5175; ^b,d^ *p* = 0.0066; ^c,d^ *p* = 0.0632; ^e,f^ *p* = 0.4917; ^e,g^ *p* = 0.2039; ^f,g^ *p* = 0.1011. Statistical significance was determined using one-way ANOVA and *p* values are from TukeyHSD post-hoc test.

**Table 2:** Loss of expression of eGFP from peGFP pre-assembled into chromatin containing unmodified histones, or H3K9,14Q acetyl-mimics.

Chromatin State of peGFP	% Loss of eGFP+ Cells/ Generation ^a^	% Loss of Fluorescence Intensity/ Generation ^a^
Unassembled	10.6 ± 0 ^b^	9.4 ± 1.3 ^e^
H3	7.7 ± 1 ^c^	9.1 ± 1.8 ^f^
H3K9,14Q	6.0 ± 0 ^d^	8.3 ± 1.8 ^g^

^a^ *n* = 4; ^b,c^ *p* = 0.0012; ^b,d^ *p* = 0.0008; ^c,d^ *p* = 0.0141; ^e,f^ *p* = 0.7808; ^e,g^ *p* = 0.3697; ^f,g^ *p* = 0.5706. Statistical significance and *p* values were determined as in Table 1.

**Table 3:** Loss of expression of eGFP from reporter plasmids pre-assembled into chromatin containing unmodified histones.

Pre-Assembled with Unmodified Histones	% Loss of eGFP+ Cells/Generation ^a^	% Loss of eGFP Fluorescence/Generation ^a^
peGFP	11.3 ± 1 ^b^	9.1 ± 2 ^e^
pW601-eGFP	5.3 ± 0 ^c^	2.0 ± 1 ^f^
pW601-100b-eGFP	9.8 ± 0 ^d^	9.5 ± 0 ^g^

^a^ *n* = 4; ^b,c^ *p* = 0.0004; ^b,d^ *p* = 0.0928; ^c,d^ *p* = 0.0002; ^e,f^ *p* = 0.0015; ^e,g^ *p* = 0.6850; ^f,g^ *p* = 0.0005. Statistical significance and *p* values were determined in Table 1.

**Table 4:** Loss of expression of eGFP from reporter plasmids pre-assembled into chromatin containing H4K16Q.

Pre-Assembled with H4K16Q	% Loss of eGFP+ Cells/Generation ^a^	% Loss of eGFP Fluorescence/Generation ^a^
peGFP	11.2 ±1 ^b^	8.4 ± 0 ^e^
pW601-eGFP	6.7 ± 0 ^c^	1 ± 3.8 ^f^
pW601-100b-eGFP	12.1 ± 0 ^d^	9.1 ± 1.8 ^g^

^a^ *n* = 4; ^b,c^ *p* = 0.0005; ^b,d^ *p* = 0.3894; ^c,d^ *p* = 0.0001; ^e,*f*^ *p* = 0.0155; ^e,g^ *p* = 0.5092; ^f,g^ *p* = 0.0082. Statistical significance and *p* values were determined as described in Table 1.

## Data Availability

The original contributions presented in this study are included in the article. Materials are available upon request.

## Supplementary Materials

The following supporting information can be downloaded at: https://www.mdpi.com/article/10.3390/genes15091232/s1, Figure S1: The human EF1α promoter driving expression of the eGFP reporter gene; Figure S2: Representative analysis of unassembled plasmid DNA or plasmids pre-assembled with unmodified histone octamers; Figure S3: Representative fluorescence microscopy images and representative flow cytometry plots; Figure S4: Impact of 100 bp spacer on transcription; Figure S5: The impact of pre-assembled nucleosomes containing H4K16Q relative to unmodified histones (H4) within plasmid constructs. References [53,54,58,59,61,67,68,70] are cited in the Supplementary Materials.
