# Supplementary material for: Impacts of Nucleosome Positioning Elements and Pre-Assembled Chromatin States on Expression and Retention of Transgenes
Source: Genes (Basel). 2024 Sep 21;15(9):1232. doi: 10.3390/genes15091232 (PMC11431089; doi:10.3390/genes15091232)
Supplement: Supplementary file 1 [file genes-15-01232-s001.zip › genes-3192765-supplementary.pdf]

A

HindIII EcoRV

5' GCAAGCTTCATTCGAGCTCGGTACCCGGGGATCCGATATCGAAGACTCTAAGTACTTTCAATACA***TGCACAGGATGTATATATCTGACACGTGCCTGGAGAC***

TAGGGAGTAATCCCCTTGGCGGTTAAAACGCGGGGGACAGCGCGTACGTGCGTTTAAGCGGTGCTAGAGCTGTCTACGACCAATTGAGCGGCCTCGGC

ACCGGGATTGTCCAGGGCGGCCGCGTAAGTACTTTCAATACATGCACAGGATGTATATATCTGACACGTGCCTGGAGACTAGGGAGTAATCCCCTTGGCG

GTTAAAACGCGGGGGACAGCGCGTACGTGCGTTTAAGCGGTGCTAGAGCTGTCTACGACCAATTGAGCGGCCTCGGCACCGGGATTGTCCAGGGCGG

CCGCGTAAGTACTTTCAATACATGCACAGGATGTATATATCTGACACGTGCCTGGAGACTAGGGAGTAATCCCCTTGGCGGTTAAAACGCGGGGGACAG

CGCGTACGTGCGTTTAAGCGGTGCTAGAGCTGTCTACGACCAATTGAGCGGCCTCGGCACCGGGATTGTCCAGGGCGGCCGCGTAAGTACTTTCAATAC

ATGCACAGGATGTATATATCTGACACGTGCCTGGAGACTAGGGAGTAATCCCCTTGGCGGTTAAAACGCGGGGGACAGCGCGTACGTGCGTTTAAGCGG

EcoRV

TGCTAGAGCTGTCTACGACCAATTGAGCGGCCTCGGCACCGGGATTGTCCAGGGCGGCCGCGTAAGACGTCTTCGATATCCTGCATAATGCATCTGGCA

SbfI HindIII

CGACAGGTTTCCCGACTGGAAAGCGGGCAGTGAGCGCAACGCAATTAATGTGAGTTAGCTCACTCATTAGGCACCCCAGGCCTGCAGGCATGCAAGGGC

ATGCAAGCTTTCATCGCGTGAGGCTCCGGTGCCCGTCAGTGGGCAGAGCGCACATCGCCCACAGTCCCCGAGAAGTTGGGGGGAGGGGTCGGCAATTG

EFP1 EFP2

AACCGGTGCCTAGAGAAGGTGGCGCGGGGTAAACTGGGAAAGTGATGTCGTGTACTGGCTCCGCCTTTTTCCCGAGGGTGGGGGAGAACCG**TATATAA**

* Exon 1

TGCAGTAGTCGCCGTGAACGTTCTTTTTCGCAACGGGTTTGCCGCCAGAACACAGGTAAGTGCCGTGTGTGGTTCCCGCGGGCCTGGCCTCTTTACGGG

TTATGGCCCTTGCGTGCCTTGAATTACTTCCACGCCCCTGGCTGCAGTACGTGATTCTTGATCCCGAGCTTCGGGTTGGAAGTGGGTGGGAGAGTTCGAG

GCCTTGCGCTTAAGGAGCCCCTTCGCCTCGTGCTTGAGTTGAGGCCTGGCCTGGGCGCTGGGGCCGCCGCGTGCGAATCTGGTGGCACCTTCGCGCCT

GTCTCGCTGCTTTCGATAAGTCTCTAGCCATTTAAAATTTTTGATGACCTGCTGCGACGCTTTTTTTCTGGCAAGATAGTCTTGTAAATGCGGGCCAAGATCT

Sp1 Sp1

GCACACTGGTATTTCGGTTTTTGGGGCCGCGGGCGGCGACGGGGCCCGTGCGTCCCAGCGCACATGTTCGGCGAGGCGGGGCCTGCGAGCGCGGCCA

Sp1 Sp1

CCGAGAATCGGACGGGGGTAGTCTCAAGCTGGCCGGCCTGCTCTGGTGCCTGGCCTCGCGCCGCCGTGTATCGCCCCGCCCTGGGCGGCAAGGCTGG

Sp1

CCCGGTCGGCACCAGTTGCGTGAGCGGAAAGATGGCCGCTTCCCGGCCCTGCTGCAGGGAGCTCAAAATGGAGGACGCGGCGCTCGGGAGAGCGGGC

Ap1

GGGTGAGTCACCCACACAAAGGAAAAGGGCCTTTCCGTCCTCAGCCGTCGCTTCATGTGACTCCACGGAGTACCGGGCGCCGTCCAGGCACCTCGATTA

GTTCTCGAGCTTTTGGAGTACGTCGTCTTTAGGTTGGGGGGAGGGGTTTTATGCGATGGAGTTTCCCCACACTGAGTGGGTGGAGACTGAAGTTAGGCCA

Exon 2

GCTTGGCACTTGATGTAATTCTCCTTGGAATTTGCCCTTTTTGAGTTTGGATCTTGGTTCATTCTCAAGCCTCAGACAGTGGTTCAAAGTTTTTTTCTTCCATT

TCAGGTGTCGTGAGGAATTCTGCAGTCGACGGTACCGCGGGCCCGGGATCCACCGGTCGCCACC**ATG 3'**

B 5' CCTGGGGTGCCTAATGAGTGAGCTAACTCACATTAATTGCGTTGCGCTCACTGCCCGCTTTCCAGTCGGGAAACCTGTCGTGCCAGCTGCATTA 3'

Figure S1. The human EF1α promoter driving expression of the eGFP reporter gene. (**a**) The array of four direct repeats of the 177 Widom601 nucleosome positioning sequence [1,2] in pW601-eGFP plasmid is shown in bold italics. The TATA box is in a solid box. The two untranslated exons (Exon 1 and Exon 2) separated by Intron-A of the EF1α promoter are shown in dotted boxes. Binding sites for transcription factors (EFP1, EFP2, Sp1 and Ap1) are noted and underlined [3,4]. The transcription start site is shown by asterisk. The translation start site of eGFP is noted at the 3′ end of the sequence (ATG in bold); (**b**) Sequence of the 100 bp fragment amplified from pUC19 DNA and cloned into the SbfI site between the Widom601 sequence and the EF1α promoter in plasmid pW601-100b-eGFP.

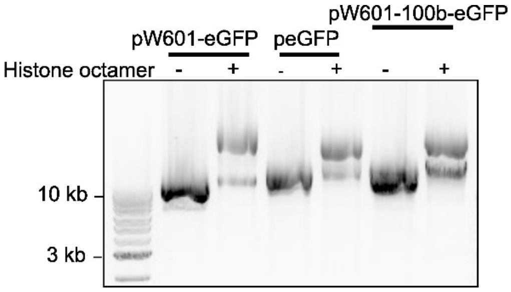

Figure S2. Representative analysis of unassembled plasmid DNA or plasmids pre-assembled with unmodified histone octamers. Samples containing 500 ng of plasmid DNA were evaluated on a 0.8% agarose gel in TBE buffer containing 89 mM Tris base, 89 mM Borate and 2 mM Na_2EDTA. All detectable plasmid DNA (~98%) had been assembled into chromatin, based on the ~10 ng limit of detection of DNA by ethidium bromide staining. Chromatin assembly was performed as described in our previous studies [5,6].

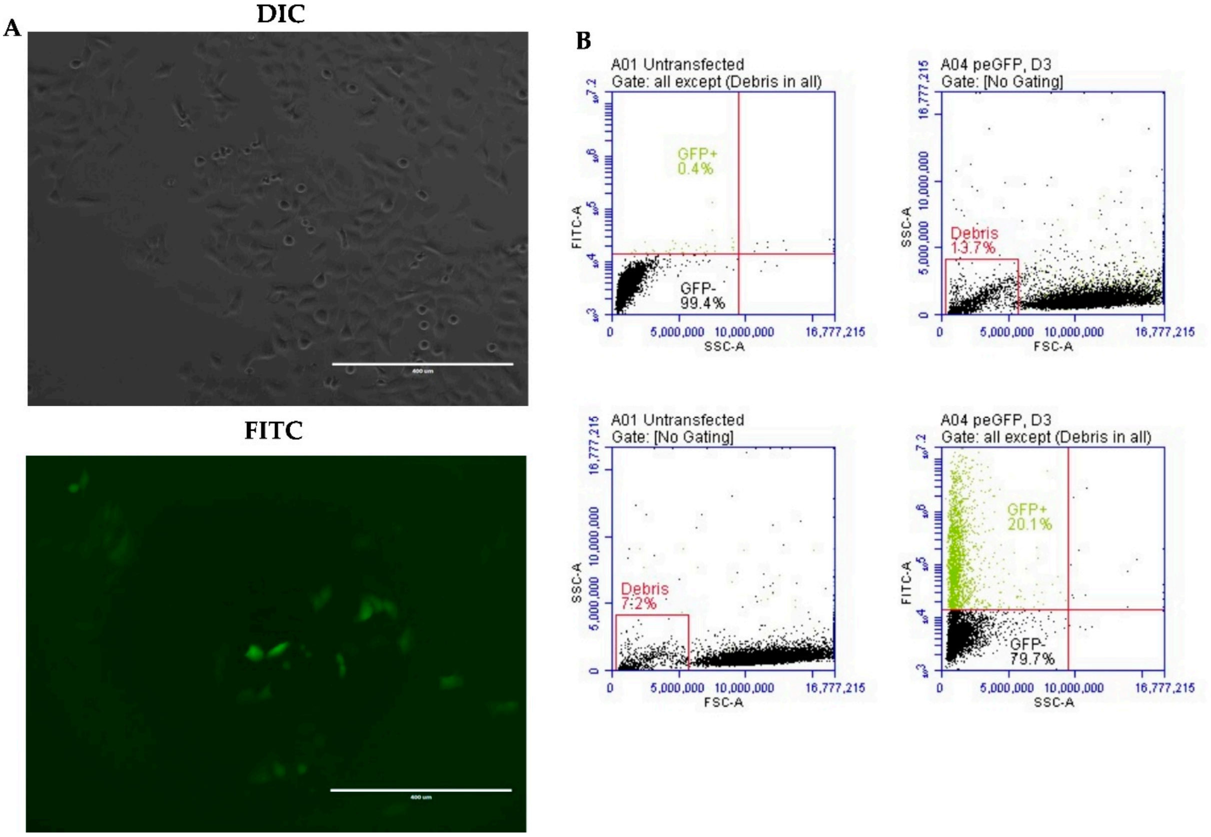

Figure S3. Representative fluorescence microscopy images and representative flow cytometry plots. (**a**) 143B cells transfected with unassembled peGFP were imaged at 72 h post-transfection under sterile live cell-imaging conditions using the EVOS FL inverted fluorescence microscope (Life Technologies). Images were taken at 10X magnification. Scale bar equals 400 µm; (**b**) Untransfected 143B cells (left panels) or 143B cells transfected with unassembled peGFP (right panels) were analyzed by flow cytometry at 72 h post-transfection. Cells were trypsinized and $1x10^5$ viable cells were isolated, washed in 1X PBS, and resuspended in 200 µl 1X PBS as described previously. Side scatter (SCC-A) and forward scatter (FSC-A) data were used to assess cell size and complexity. After thresholding to remove "Debris" (top panels) ten thousand events were analyzed, and eGFP+ cells were detected through the FL-1 488 nm channel (FITC-A) against the side scatter (SCC-A). Mean fluorescence intensity of $1x10^4$ was set as the baseline for the FITC-A channel. Untransfected control cells had 0.4% eGFP+ cells above baseline, while those cells transfected with peGFP had a 20.1% eGFP+ cells at three days post-transfection. The fluorescence intensity of eGFP above baseline ranged from $1x10^4$ to $1x10^7$ (arbitrary units)_(Figure S3B, bottom panels), and was used to calculate the mean intensity of fluorescence signal from the eGFP+ cells (see Materials and Methods).

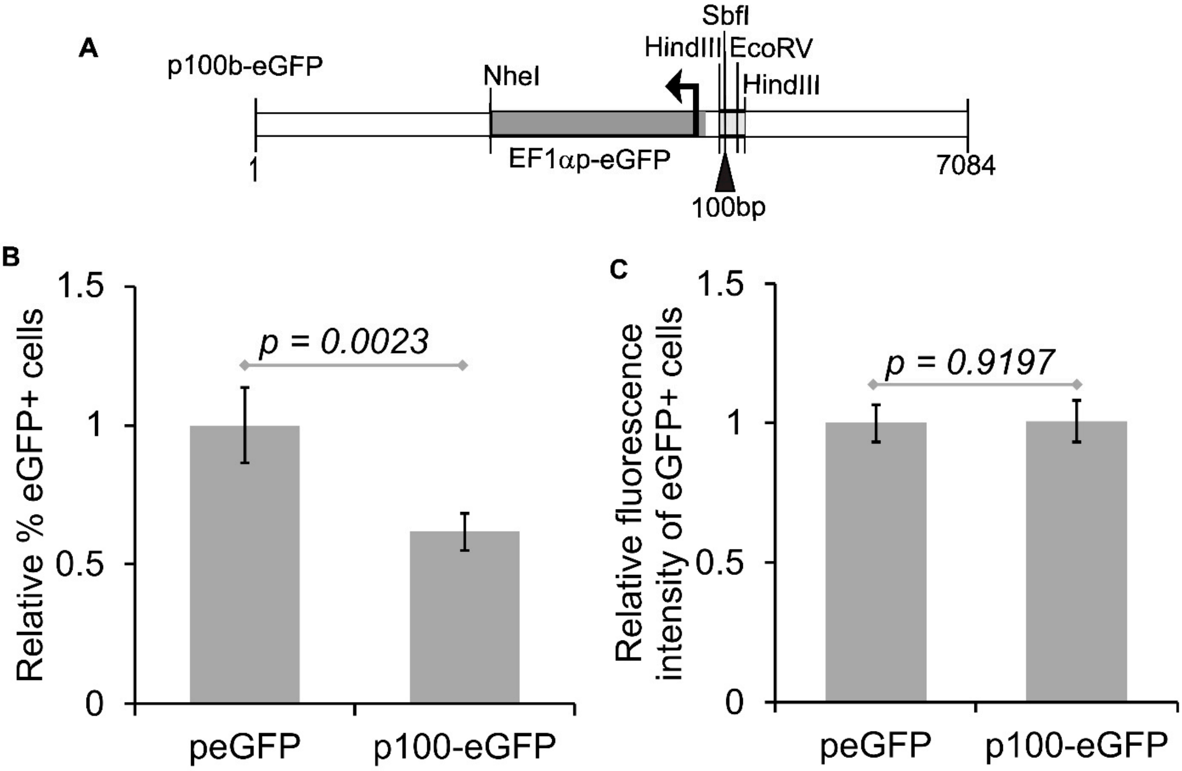

Figure S4: Impact of 100 bp spacer on transcription. (**a**) Map of p100-eGFP. (**b**) Percent eGFP+ cells at D3 were calculated for samples transfected with the indicated "naked" plasmids, and then normalized relative to percent eGFP+ cells at D3 from cells transfected with peGFP, which was set to 1 (Mean ± STD, n = 4); (**c**) Mean fluorescence intensity of eGFP+ subpopulations in Figure S2B were calculated and then normalized relative to the mean fluorescence intensity at D3 of cells transfected with peGFP, which was set to 1 (Mean ± STD, n = 4). Statistical significance was determined using One-way Anova [7] and p values are from TukeyHSD post-hoc test [8].

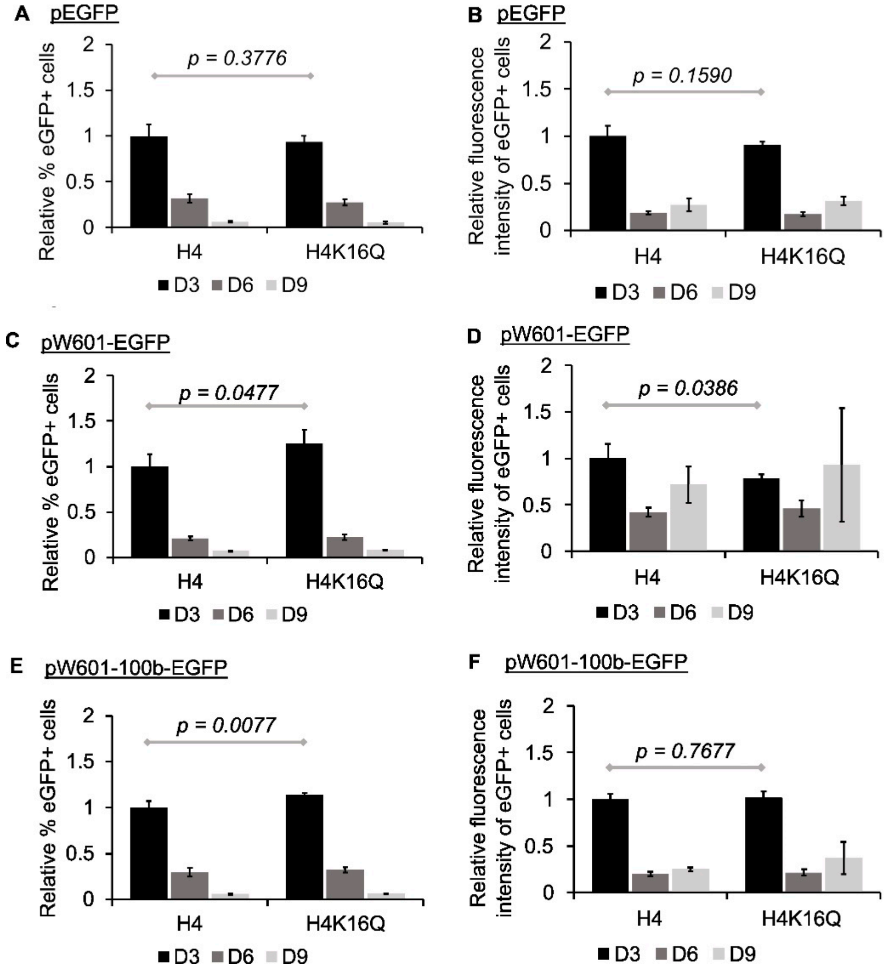

Figure S5: The impact of pre-assembled nucleosomes containing H4K16Q relative to unmodified histones (H4) within plasmid constructs. Re-analysis of data from experiment shown in Figure 4. (**a,c&e**) Percent eGFP+ cells from H4K16Q-containing chromatin normalized relative to percent eGFP+ cells from H4-containing chromatin on the indicated plasmids; (**b,d&f**) MFI of the eGFP+ subpopulation from H4K16Q-containing chromatin normalized relative to MFI of the eGFP+ subpopulation from H4-containing chromatin on the indicated plasmids. Statistical significance was determined as in Figure 2.

References

1. Lowary, P.T.; Widom, J. New DNA sequence rules for high affinity binding to histone octamer and sequence-directed nucleosome positioning. *J. Mol. Biol.* **1998**, *276*, 19-42, doi:10.1006/jmbi.1997.1494.
2. Makde, R.D.; England, J.R.; Yennawar, H.P.; Tan, S. Structure of RCC1 chromatin factor bound to the nucleosome core particle. *Nature* **2010**, *467*, 562-566, doi:10.1038/nature09321.
3. Uetsuki, T.; Naito, A.; Nagata, S.; Kaziro, Y. Isolation and Characterization of the Human Chromosomal Gene for Polypeptide Chain Elongation Factor-1α. *J. Biol. Chem.* **1989**, *264*, 5791-5798, doi:doi.org/10.1016/S0021-9258(18)83619-5.
4. Wakabayashi-Ito, N.; Nagata, S. Characterization of the regulatory elements in the promoter of the human elongation factor-1 alpha gene. *J. Biol. Chem.* **1994**, *269*, 29831-29837, doi:doi.org/10.1016/S0021-9258(18)43956-7.
5. Jimenez-Useche, I.; Yuan, C. The Effect of DNA CpG Methylation on the Dynamic Conformation of a Nucleosome. *Biophys. J.* **2012**, *103*, 2502-2512, doi:doi.org/10.1016/j.bpj.2012.11.012.
6. Nurse, N.P.; Yuan, C. Cis and trans internucleosomal interactions of H3 and H4 tails in tetranucleosomes. *Biopolymers* **2015**, *103*, 33-40, doi:doi.org/10.1002/bip.22560.
7. Bewick, V.; Cheek, L.; Ball, J. Statistics review 9: One-way analysis of variance. *Crit. Care* **2004**, *8*, 130, doi:10.1186/cc2836.
8. Abdi, H.; Williams, L.J. Tukey's honestly significant difference (HSD) test. *Encyclopedia of Research Design* **2010**, *3*, 1-5.
